# Development of a prenatal smoking cessation counseling scale for public health nurses in Japan

**DOI:** 10.18332/tid/140088

**Published:** 2021-08-02

**Authors:** Meng Li, Reiko Okamoto, Misaki Kiya, Miho Tanaka, Keiko Koide

**Affiliations:** 1 Division of Health Sciences, Osaka University Graduate School of Medicine, Osaka, Japan

**Keywords:** pregnant women, smoking cessation, counseling, public health nurses, Japan

## Abstract

**INTRODUCTION:**

This study aimed to develop a scale to measure prenatal smoking cessation counseling for Japanese public health nurses (PHNs).

**METHODS:**

A cross-sectional study was conducted via an anonymous, self-administered questionnaire. The sample included 1933 PHNs working in 424 municipal health centers nationwide, which were randomly selected. We created the draft scale based on semi-structured interviews, previous studies, and preliminary survey. Additionally, we conducted back translation for English version of the draft scale to be applicable in English countries. The analytic strategy consisted of item analysis, exploratory factor analysis, and differentiation by ‘known groups’.

**RESULTS:**

A total of 550 responses (28.5%) were included in the analysis. Most of the respondents were female (98.2%) and the mean age was 37.5±9.37 years. In the exploratory factor analysis, two factors were extracted and the factor loadings for all items were greater than 0.40. The first factor with eleven items was named as ‘basic counseling’ and the second factor with seven items was named as ‘advanced counseling’. The Cronbach’s alpha of the scale was 0.918, and the cumulative contribution was 44.908%. Multiple comparisons by experience years working as a PHN revealed significant differences in the scale and two factors.

**CONCLUSIONS:**

In this study, we initially developed the prenatal smoking cessation counseling scale for Japanese PHNs, and the reliability and validity of the scale were considered to be acceptable.

## INTRODUCTION

Maternal smoking during pregnancy is a modifiable risk factor influencing maternal and fetal health^1^. Maternal smoking and exposure to secondhand smoke (SHS) during pregnancy are associated with increased risks for ectopic pregnancy, preterm premature rupture of the membrane, placenta previa, placental abruption, miscarriage, stillbirth, preterm birth (<37 weeks gestation), low birthweight (<2500 g), small for gestational age, and congenital anomalies such as cleft lip^2-10^. After birth, the risk of sudden infant death syndrome (SIDS) is higher among the offspring of women who smoked during or after pregnancy^11^. According to a recent systematic review, global prevalence of maternal smoking during pregnancy is estimated to be 1.7%. The prevalence of smoking during pregnancy is higher than 10% in 29 of 174 countries and more than 20% in 12 countries^12^.

In Japan, the prevalence of maternal smoking during pregnancy is estimated to be between 5.1% and 7.5%^12^, and the prevalence of paternal smoking is reported to be 47% in the first trimester and 46% in the second and third trimesters^13^. In light of this troubling fact, the Japanese government has enacted several national-level legal regulations and policies, such as ‘Health Japan 21’ and ‘Health Promotion Act’ where reducing maternal smoking and SHS exposure during pregnancy were recorded^14,15^. At the prefectural and municipal levels, local governments have implemented a series of measures such as the Kanagawa prefecture anti-smoking ordinance according to national regulations and policies^16^. Of these, previous study has reported that approximately 80% of the professionals who play a central role in prefectural and municipal measures on tobacco control are PHNs^16^. Furthermore, in municipal level, PHNs working in health centers engage with community members with a wide range of health levels including infants, young children, elderly people, pregnant women, and people with disabilities^17^. Therefore, PHNs play an important role in preventing maternal smoking and SHS exposure during pregnancy in municipalities. Additionally, previous study has reported that PHNs can reduce maternal smoking and SHS exposure during pregnancy because they have many opportunities, such as the issuance of maternal and child health handbook, telephone counseling, classroom sessions, home visits, and health checkups of infants, to engage with pregnant women^18^. Psychological intervention has been recommended as the first line of treatment to help pregnant women to quit smoking^19,20^. The World Health Organization (WHO) has recommended that healthcare providers routinely offer advice and psychological interventions for tobacco cessation to pregnant women who are either current tobacco users or recent tobacco quitters^1^. As a brief psychological intervention method, the 5As model (Ask, Advise, Assess, Assist, and Arrange follow-up)^20^ has been recommended to be used at the first antenatal appointment and subsequent appointments, in previous literature^21^. Japanese guidelines for smoking cessation also recommend healthcare providers to adopt the 5As model for smoking cessation in general practice settings such as outpatient care and medical examination^22^. Additionally, previous literature has reported that pregnancy is an excellent opportunity to quit smoking because patients visit the clinic regularly and smoking cessation may be motivated effectively^1,22^. Overall, psychological interventions should be prioritized to help women to quit smoking, and the 5As model can be applicable for smoking cessation intervention during pregnancy.

In Japan, regarding psychological interventions for smoking cessation, previous studies have mainly centered on nurses’ behavioral counseling in Japanese smoking cessation therapy. These studies have reported that nurses’ behavioral counseling skills such as providing patients with reasons to quit, helping patients to cope with recovery symptoms, and managing the patient’s weight gain, can help smokers to quit smoking by reinforcing patients’ selfefficacy and controlling the strength of the patients’ craving^23-25^. Additionally, previous study has reported that most nurses felt that although they assessed and documented the tobacco status of cancer patients, they were not successful in providing cessation advice, assessing patient readiness to quit, and providing individualized information on the harmful effects of tobacco use in cancer and general hospitals^26^. Overall, nurses’ behavioral counseling has been used for smoking cessation interventions, whereas the levels of counseling skills are different among Japanese nurses.

To date, although PHNs are expected to play an important role in preventing maternal smoking and SHS exposure during pregnancy, there are no scales to measure prenatal smoking cessation counseling among Japanese PHNs. Therefore, this study aimed to develop a scale to measure prenatal smoking cessation counseling for Japanese PHNs. The scale will be expected to improve the behavioral counseling skills by measuring prenatal smoking cessation counseling with this scale.

## METHODS

### Design and sample

We conducted a cross-sectional study from 1 January to 15 February 2020 via an anonymous, selfadministered questionnaire. The sample included 1933 PHNs working in 424 municipal health centers nationwide. The study was approved by the Ethical Committee of Observation Research at Osaka University Hospital (Approval No: 19308, 28 November 2019).

### Creating the draft scale

Creation of the draft scale was divided into five stages. First, we discussed the feasibility of developing the scale using the 5As model among the researchers. Second, we conducted semi-structured interviews with 10 PHNs who had abundant experience in maternal and child health, to obtain their opinions for prenatal smoking cessation counseling. Third, we extracted 68 items from the semi-structured interviews and previous studies^1,19,21,22,27-31^, and subsequently categorized them according to the 5As model. Fourth, we conducted a preliminary survey of 27 PHNs to obtain opinions on the contents and wordings, and then completed the draft scale based on the opinions and our considerations. The draft scale included 36 items rated on a 6-point Likert scale (0 to 5 representing ‘strongly disagree’ to ‘strongly agree’).

After completing the cross-sectional survey, we excluded five items in the analysis of scale development, because these items were already recorded in the pregnancy notification form which meant PHNs have implemented these contents in the usual work.

### Back translation for English version of the draft scale

To be available in English for other countries, we conducted back translation for the English version of the draft scale. The back translation process was divided into five stages. First, we invited two native English translators who were very familiar with Japanese translations to conduct independent forward translation on the draft scale. Second, we compared and discussed the two forward translations and the draft scale among the researchers, and subsequently created an integrated forward translation version. Third, we invited two native Japanese translators who were very familiar with English translations to carry out independent back-translation on the integrated forward translation version. Fourth, we compared and discussed the two back-translated versions and the original scale draft among the researchers and then compiled an integrated back translation version of the scale draft. Finally, we invited seven native English speakers to check the wording, grammar and translation congruence of English on the integrated back translation version to finalize the English version of the scale draft.

### Data collection

First we randomly selected 1933 PHNs from 424 municipal health centers nationwide according to the number of full-time PHNs from Official Statistics of Japan in e-Stat^32^. Second, we searched the addresses of 424 municipal health centers on the internet, and sent an envelope including the request for participant cooperation, the return envelope, and the questionnaire, to 424 municipal health centers. Third, to increase the response rate, we sent a reminder in approximately one month after the questionnaire was mailed out.

### Statistical analysis

The statistical analysis consisted of item analysis, exploratory factor analysis, and differentiation by ‘known groups’.

For item analysis, the average and standard deviation of the 31 item responses were firstly computed. In examining response biases, the ceiling and floor effects were assessed by the average value ± 1 SD for each item, and the items that exceeded the maximum value or minimum value were excluded. To avoid duplicate items, the Pearson’s correlation coefficient with r≥0.75 was considered to be similar in the semantic contents, and one item was excluded^33^. To verify the discriminative power of each item, we used the quartile method to divide them into the top quartile group (good group) and the low quartile group (poor group). Subsequently, we compared the average values for each item’s scores between the two groups. The items that had no significant difference were excluded. Additionally, we performed the inter-item and total-item analysis to determine the correlations between scale items as well as the correlations between each item and the sum score of scale items, and those with correlation coefficient r<0.3 were excluded^34^.

For exploratory factor analysis, we performed the principal component method with promax rotation. The items with factor loadings ≥0.40 in one of the extracted factors were collected together to construct the common factor^34^. The items that did not meet the criteria were excluded, and the analysis was repeated. Finally, the extracted factor was named to reflect the included items best. For reliability, Cronbach’s alpha coefficients were used to verify internal consistency reliability. For differentiation by ‘known groups’, we performed one-way analysis of variance (ANOVA) for the relationship between years of experience as a PHN and the factors^34^.

Data analysis was performed using SPSS Statistics 25.0 for Microsoft Windows (IBM Japan, Tokyo, Japan). For all analyses, p<0.05 was considered statistically significant.

## RESULTS

Of the questionnaires mailed to PHNs, 599 (31.0%) responses were received of which 49 were excluded because they missed an answer or had multiple answers for the questions. Finally, 550 responses (28.5%) were included in the analysis.

Characteristics of the respondents are summarized in Table 1. Most of the respondents were female (98.2%) and the mean age was 37.5±9.37 years. The average years working as a PHN was 12.5±9.47. More than half (58.9%) were staff level, followed by assistant managerial level (34.5%). More than half (55.5%) had a 4-year university education, followed by those who had completed vocational college (27.8%) and junior college (15.6%).

**Table 1:** Characteristics of respondents included in the analysis (N=550)

*Characteristics*	*n*	*%*	*Mean*	*SD*
**Gender**				
Female	540	98.2		
Male	10	1.8		
**Age** (years)			37.5	9.37
≤29	141	25.6		
30–39	170	30.9		
40–49	173	31.5		
≥50	66	12.0		
**Experience as a public health nurse** (years)			12.5	9.47
≤5	185	33.6		
6–15	159	28.9		
≥16	206	37.5		
**Work position**				
Staff	324	58.9		
Assistant managerial level	190	34.5		
Managerial level	36	6.5		
**Educational level**				
Vocational college	153	27.8		
Junior college	86	15.6		
University (4 years)	305	55.5		
Master’s program	6	1.1		

The results of the item analysis are summarized in the Supplementary file. Six items (items 1, 2, 14, 17, 18 and 20) showed the ceiling effect and item 12 showed the floor effect. Thus, these items were excluded. Nine pairs (items 5–6, 25–26, 27–28, 28– 29, 29–30, 30–31, 27–29, 27–30 and 28–30) were considered to be similar in the semantic contents with the correlation coefficient ≥0.75. Thus, five items (item 5, 25, 28, 29, and 30) were excluded. The mean value of each item between the first and fourth quartile groups had a statistically significant difference (p<0.001). Moreover, the correlation coefficient between scale items ranged 0.497–0.712 and the correlation coefficient between each item and the total scale ranged 0.556–0.752.

Exploratory factor analysis was conducted on the remaining 19 items. Two factors were extracted based on the screen plot criteria, and one item (item 13) was excluded because of low factor loading (<0.40). The factor loadings for all items were greater than 0.40. The first factor with eleven items was denoted ‘basic counseling’ because the items included basic counseling skills such as explaining the harmful effects of smoking and SHS, and encouraging the expectant mother to quit smoking using anti-smoking materials like pamphlets, charts, and photos. Similarly, the second factor with seven items was denoted ‘advanced counseling’ because the items included advanced counseling skills such as providing the methods of quitting smoking and dealing with withdrawal symptoms. Regarding contributing factors, 39.497% of the variance was explained by the first factor, the second factor explained 5.411%, and the cumulative contribution was 44.908%. The Cronbach’s alpha coefficient of the scale was 0.918. In the factors, the Cronbach’s alpha coefficient of the first factor was 0.864 and for the second factor it was 0.891. The correlation between the two factors was 0.711 (Table 2).

**Table 2:** Exploratory factor analysis of the prenatal smoking cessation counseling scale (N=550)

*Item*	*Factor 1: Basic counseling*	*Factor 1*	*Factor 2*
15	If the expectant mother lives with a smoker, I explain the benefits of household members abstaining from smoking and get the expectant mother to encourage them to quit smoking.	0.719	-0.062
16	If the expectant mother lives with a smoker, I explain methods by which the expectant mother can avoid secondhand smoke.	0.698	-0.050
10	I confirm the expectant mother’s intention to quit smoking in each interview.	0.643	-0.079
7	I use interview techniques such as active listening, acceptance, empathy, and motivational interviewing to encourage the expectant mother to quit smoking.	0.640	0.027
8	I encourage the expectant mother to quit smoking based on her personal traits (personality, medical history, reasons for smoking, etc.).	0.613	0.020
19	If the expectant mother lives with a former smoker who has quit smoking, I compliment household members on quitting smoking and get the expectant mother to ask them to keep it up.	0.584	0.063
3	I explain the harmful effects of the toxic substances contained in tobacco (nicotine, tar, carbon monoxide, etc.).	0.546	0.029
27	I confirm the smoking abstinence status of the expectant mother and her household members.	0.539	0.054
11	I confirm the expectant mother’s stages of behavioral change toward quitting smoking in each interview.	0.539	0.160
9	I use anti-smoking materials like pamphlets, charts, and photos to encourage the expectant mother to quit smoking.	0.463	0.094
4	I explain the expectant mother’s new role and responsibilities as a mother.	0.447	0.158
***Item***	***Factor 2: Advanced counseling***		
22	I provide information on smoking cessation outpatient services and smoking cessation clinics that are covered by medical insurance.	-0.129	0.930
21	I explain that smoking cessation treatment is ‘reliable’ and ‘not very expensive’.	-0.043	0.880
6	I explain that smoking cessation treatment increases the success rate of quitting smoking.		
23	I recommend the use of online easy-to-use smoking cessation support services and programs.	-.086	.666
26	I provide some methods on how to deal with withdrawal symptoms after quitting smoking (irritability, headaches, sleeplessness, etc.).	-0.094	0.663
24	I recommend the expectant mother to ask for support from her family, friends, co-workers, and other acquaintances.	0.189	0.608
31	If the expectant mother is still smoking, I communicate that she can start smoking cessation treatment at any time.	0.201	0.523
**Cronbach’s alpha:** 0.918 (for the scale)		0.864	0.891
**Cumulative proportion** (%)		39.497	44.908
**Factor correlations F1**			0.711**

Principal component method with promax rotation.

** p<0.01. Full list of items given in Supplementary file.

Regarding the differentiation by ‘known groups’, multiple comparisons by experience years working as a PHN revealed significant differences between 6–15 years and ≥16 years, and ≤5 years and ≥16 years in the scale and basic counseling, and ≤5 years and 6–15 years, 6–15 years and ≥16 years, and ≤5 years and ≥16 years in advanced counseling (Table 3).

**Table 3:** Multiple comparisons by experience years working as a PHN (N=550)

	*The scale*		*Basic counseling*		*Advanced counseling*	
	*Mean±SD*	*p*	*Mean±SD*	*p*	*Mean±SD*	*p*
**Experience years working as a PHN**						
≤5	50.30±16.11	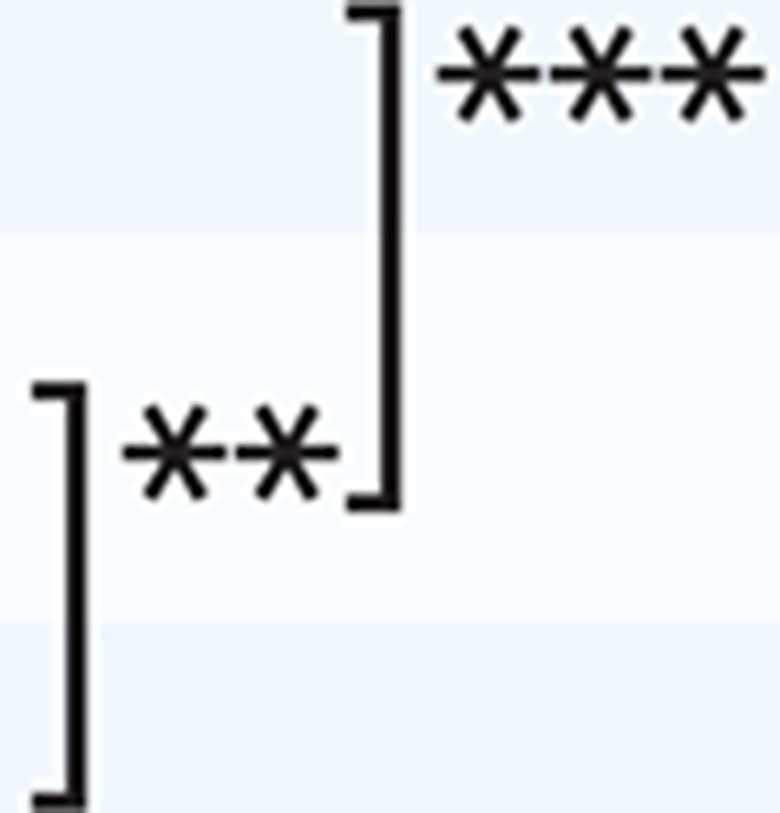	35.69±9.96	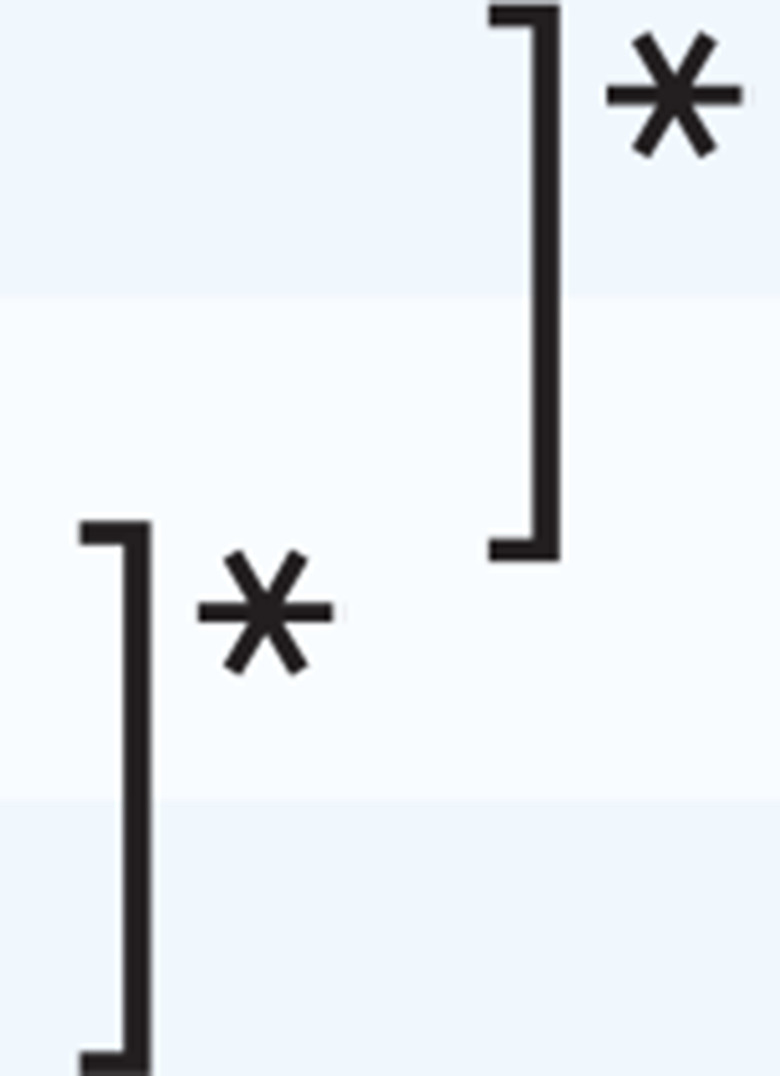	14.61±7.70	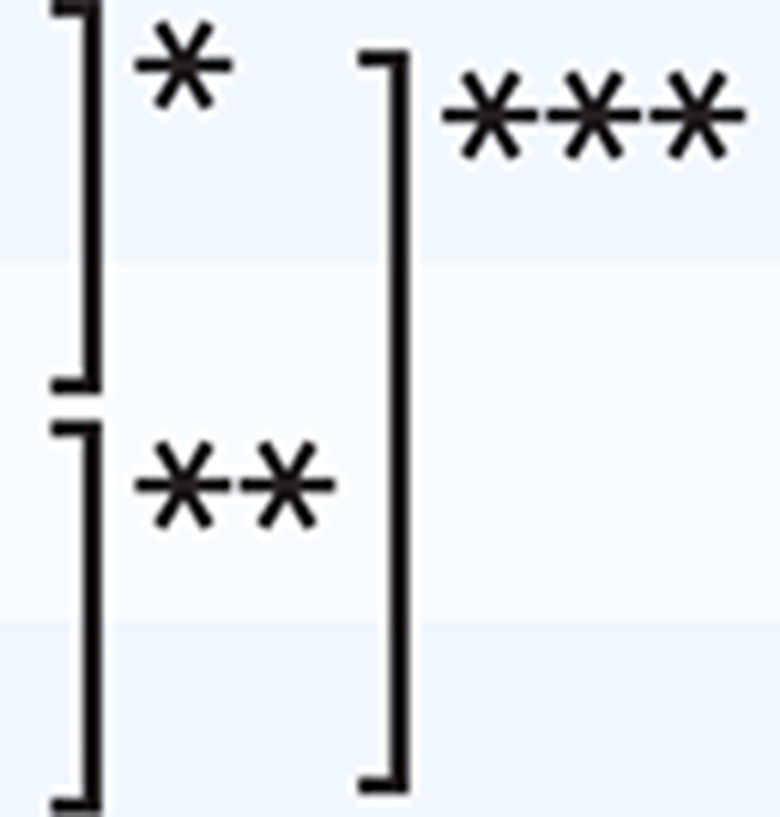
6–15	52.57±15.41		35.88±9.24		16.69±7.43	
≥16	57.56±15.76		38.24±9.24		19.33±7.75	

*p<0.05, **p<0.01, ***p<0.001.

## DISCUSSION

The 5As model has been frequently used for smoking cessation interventions in previous studies^35-42^. However, there are no scales to measure prenatal smoking cessation counseling. Therefore, the scale presented here is the first instrument to measure the prenatal smoking cessation counseling among Japanese PHNs. Regarding the reliability of the scale, Boateng et al.^34^ reported that an alpha coefficient of 0.70 has often been regarded as an acceptable threshold for reliability. However, 0.80 and 0.95 are preferred for the psychometric quality of scales. In this study, the Cronbach’s alpha coefficient of the scale was 0.918, and the two factors were both greater than 0.85. Therefore, the scale and two factors were considered to be preferred for the psychometric quality of scales.

The scale was structured with 18 items covering two factors: basic counseling and advanced counseling. The basic counseling encompassed some basic skills such as explaining the harmful effects of smoking and SHS, and encouraging the expectant mother to quit smoking using anti-smoking materials like pamphlets, charts, and photos. The advanced counseling encompassed some advanced skills such as providing the methods of quitting smoking and dealing with withdrawal symptoms. The contents were extracted from the qualitative study and previous studies^1,19,21,22,27-31^, and assessed by 27 PHNs who had extensive experience in maternal and child health. Therefore, the content validity was considered to be preferred. For construct validity, the factor loadings for all items were greater than 0.40, which met the criteria in the exploratory factor analysis^34^.

This scale was developed to measure prenatal smoking cessation counseling, and to promote nursing interventions for smoking cessation. However, previous studies reported that numerous factors were associated with nursing interventions for smoking cessation. For example, a recent narrative review grouped these factors into four conceptually similar categories including socioeconomic factors, smoking-related factors, motivational factors, and enabling factors and barriers^43^. Additionally, a previous qualitative study in Belgium reported that barriers affecting midwives and gynecologists providing smoking cessation during pregnancy were fear of provoking resistance and lack of time and communication skills regarding smoking cessation^44^. Overall, nursing interventions for prenatal smoking cessation will need to be improved based on the identified factors in future work.

There are three suggestions for scale utilization. First, the scale may be used to evaluate the effects of smoking cessation intervention training. In Japan, there are several smoking cessation intervention training programs such as Japan Smoking Cessation Training Outreach Project (J-STOP). However, there are no scales to evaluate prenatal smoking cessation counseling. Second, the contents of the scale draft may be used in primary PHN education. The contents are extracted from the qualitative study and previous literature such as Japanese guidelines for smoking cessation. Third, the perceptions and attitudes of PHNs for their supportive role in promoting prenatal smoking cessation may be improved by developing the scale.

### Strengths and limitations

This study has several limitations. First, the effective response rate in this study was only 28.5%. Thus, the sample may not be representative of PHNs nationwide. Second, the scale was developed to measure prenatal smoking cessation counseling for Japanese PHNs. However, to be available in other countries in English, we conducted back translation for the English version of the draft scale. Third, we did not make the test-retest study to ensure the reliability of the scale. Thus, the reliability of the scale will need to be evaluate in further work.

## CONCLUSIONS

A nationwide cross-sectional study was conducted via an anonymous, self-administered questionnaire. The sample included 1933 public health nurses working in 424 municipal health centers nationwide. A total of 550 responses (28.5%) were included in the analysis. Two factors were extracted, and the factor loadings for all items were greater than 0.40. The first factor with eleven items was denoted ‘basic counseling’, and the second factor with seven items was denoted ‘advanced counseling’. Cronbach’s alpha coefficient of the scale was 0.918. Multiple comparisons by experience years working as a public health nurse revealed significant differences in the scale and two factors. Therefore, the reliability and validity of the scale were considered to be acceptable. The scale will be expected to measure prenatal smoking cessation counseling and promote nursing interventions for smoking cessation.

## Data Availability

The data supporting this research is available from the authors on reasonable request.
